# Supportive eHealth Technologies and Their Effects on Physical Functioning and Quality of Life for People With Lung Cancer: Systematic Review

**DOI:** 10.2196/53015

**Published:** 2024-07-26

**Authors:** Suriya Kirkpatrick, Zoe Davey, Peter Richard Wright, Catherine Henshall

**Affiliations:** 1 School of Nursing Faculty of Health and Life Sciences Oxford Brookes University Oxford United Kingdom

**Keywords:** lung cancer, physical activity, exercise, physical functioning, mobile technology, smartphone apps, digital health, mobile phone

## Abstract

**Background:**

Despite advancements in treatment and early diagnosis, people with lung cancer are not living as long as those with other cancers. The more common symptoms of lung cancer, such as breathlessness, fatigue, and depression, can be alleviated by improving patients’ physical functioning. Therefore, good symptom management and improved health-related quality of life (HRQoL) are priorities in this patient group. However, current health care services have limited capacity to provide this support. One way to address this issue of health care resources is to empower patients to self-manage their condition using eHealth technologies.

**Objective:**

The purpose of this review was to identify and assess available research on technologies that support persons with lung cancer to improve or maintain their physical functioning, HRQoL, or both.

**Methods:**

Six databases—PubMed, Web of Science, CINAHL, MEDLINE, SPORTDiscus, and PsycINFO—were searched from January 1, 1990, to April 30, 2023. Studies were suitable for inclusion if the participants included people with lung cancer aged >18 years who had been exposed to a physical activity, exercise, or training intervention that was delivered via an electronic or web-based application with or without a comparator. Furthermore, the study had to report on the impact of the intervention on physical functioning and HRQoL. Studies that focused on telemedicine without a digital intervention were excluded. The Grading of Recommendations Assessment, Development, and Evaluation system was used to assess the quality of the included papers. Due to the heterogeneity of the studies, a narrative synthesis was undertaken.

**Results:**

This review is reported in accordance with the PRISMA (Preferred Reporting Items for Systematic Reviews and Meta-Analyses) guidelines. A total of 794 papers were initially identified through our search, of which, after screening, 8 (1%) were confirmed suitable for inclusion in the review. As 2 (25%) of the 8 papers reported on different stages of the same study, we included only 7 studies in our analysis. The studies were undertaken between 2010 and 2018 across multiple countries and aimed to develop a technology and test its feasibility or acceptance. The 7 technologies identified included web-based applications, mobile apps, and gaming consoles. The studies demonstrated impact on walking distance, muscle strength, balance, dyspnea symptoms, and cancer-related fatigue. HRQoL scores also showed improvement.

**Conclusions:**

The findings indicate that eHealth technologies can positively impact physical functioning and well-being for people with lung cancer, but there are limited studies that demonstrate the impact of these digital interventions over longer periods. None of the studies reported on the implementation or adoption of a mobile health or eHealth intervention in routine clinical practice, highlighting the need for further research in this area.

**Trial Registration:**

PROSPERO CRD42023414094; https://tinyurl.com/39hhbwyx

## Introduction

### Background

Lung cancer is the most prevalent cancer globally, with 2.21 million new cases being diagnosed in 2020; this is anticipated to increase to 3.8 million by 2050 [1]. Lung cancer also accounts for the highest number of cancer-related deaths across all cancer types [2].

In the United Kingdom, approximately 48,500 new lung cancer cases are diagnosed per year [3]. The incidence of lung cancer strongly correlates with age, peaking among older individuals. In the United Kingdom, from 2016 to 2018, >44% of new cases annually were in those aged ≥75 years. Age-specific incidence rates rise sharply from around ages 45 to 49 years, reaching a peak in women aged 75 to 79 years and men aged 85 to 89 years, and then decline in older age groups. Women typically have lower incidence rates than men, particularly evident at age ≥90 years, where the rate in women is half that of men [3]. One-year survival rates have almost doubled since the 1970s due to early diagnosis and improved treatments. However, lung cancer survival rates at 5 and 10 years have not improved as much as those for other cancers [3].

For people living with lung cancer, it is imperative that supportive care needs, which are central to patient-centered care [4], are addressed promptly because their condition is associated with a high symptom burden and high levels of unmet needs throughout the disease trajectory [5]. In addition, approximately two-thirds of people with lung cancer have at least 1 other preexisting health condition, and up to half have ≥2 preexisting health conditions [6]. Addressing their supportive care needs will contribute to efficient use of health care resources and minimize hospital admissions. If not managed effectively, this could negatively impact patient outcomes, including physical functioning, psychological well-being, and health-related quality of life (HRQoL) [4].

Common symptoms of lung cancer include fatigue, breathlessness, and depression—all of which can be alleviated by exercise interventions [7]. More generally, other positive implications for people with cancer undertaking physical activity include improvements in HRQoL [8-11], lung function [8,10], sleep [8,12], immune function and markers [8,13], mood [8,9], bone strength [8,14], and muscle mass [8,15], as well as decreased cancer cell proliferation [8,13]. However, less than one-third of people with lung cancer meet recommended exercise guidelines to reduce time spent sedentary, increase strength- and balance-building activities, and undertake 150 minutes of aerobic activity per week [16].

Self-management practices, including those with an exercise component, can help patients with cancer to regain health and fitness, reduce side effects from treatment and symptoms of the disease, relax the mind and body, enhance HRQoL, and regain a sense of normality [17]. More recently, the National Institute for Health and Care Excellence has recognized exercise as a first-line treatment within health care and holistic disease management [18]. In the absence of a robust national rehabilitation system, there is pressure for self-management support to be integrated into routine cancer care [19]. However, patient adherence to rehabilitation programs delivered at hospital outpatient centers can be low due to the required travel and associated socioeconomic factors [20]. Furthermore, studies have demonstrated that home-based rehabilitation improves patient adherence and HRQoL [21].

Web-based interventions have grown in popularity in recent years. These interventions enable the user to independently navigate a recommended online program that is operated via a website with the aim to create positive changes in health and well-being [22]. Government organizations are actively trying to transition in-person activities to web-based platforms [23]. After the COVID-19–related lockdown, this gained renewed prominence because the advantages of digital technology became increasingly evident. Furthermore, earlier reviews have identified several mobile and electronic apps designed to support various stages of the cancer continuum ranging from prevention to survivorship [24]. The use of mobile health (mHealth) and eHealth technologies, such as wearables and activity trackers as well as apps and web-based programs that can be accessed via smartphones and tablets, provide new methods for educating, monitoring, and supporting patients with chronic conditions and cancer. The World Health Organization recognizes the potential of mHealth and eHealth interventions to support health care delivery [25]. These interventions can assist patients in self-managing their health behaviors and are considered feasible, acceptable, and effective approaches to providing supportive care [26,27].

There is a growing body of evidence to support technology interventions in health care, and this is supported within the UK National Health Service Long Term Plan [28]. Nevertheless, evidence-based mHealth and eHealth interventions to enhance exercise and physical activity for people with lung cancer remain uncommon. Furthermore, of the cancer-related apps that are available, a limited number adopt a personalized approach to physical activity and exercise that accommodates patients’ preferences.

### Aim

This study aims to identify the mHealth and eHealth technologies that have been developed to support people with lung cancer to improve or maintain physical functioning and enhance their HRQoL.

### Objectives

The primary objective of the review was to determine whether any of the mHealth or eHealth technologies identified impacted the physical functioning and HRQoL of people with lung cancer.

The secondary objectives were to assess the demand on clinician time; evaluate the acceptability of the intervention to patients, carers, and health care professionals; investigate user satisfaction with the technology; identify security features (clinical safety, data protection, and technical security); and examine the cost impact of the mHealth or eHealth app or intervention.

## Methods

### Design

This review was prospectively registered with PROSPERO (CRD42023414094) and is reported in accordance with the PRISMA (Preferred Reporting Items for Systematic Reviews and Meta-Analyses) guidelines to improve the quality of the review and ensure transparency at all stages.

### Inclusion Criteria

#### Types of Studies

We included all primary research studies, without study design or publication status limitations or language or geographic area restrictions. Case studies were also included. Reference lists of systematic reviews were cross-checked to identify any potential studies for inclusion. We limited the search to studies published after January 1, 1990, because internet interventions did not exist before this date [29].

#### Population

We included studies that were undertaken with adults (aged >18 y) who were diagnosed with lung cancer, regardless of the stage of their disease, treatment allocation, sex, or where they received care.

#### Intervention

Study participants in the included studies must have been exposed to a physical activity, exercise, or training intervention that was delivered via an electronic or web-based application with or without a comparator.

#### Outcome Measures

Studies were included if they reported on the impact of the digital intervention on physical function or HRQoL or both physical functioning and HRQoL using any validated measure. We included studies that measured impact at ≥1 time points.

### Exclusion Criteria

The following studies were excluded: (1) cancer studies in which the total number of participants with lung cancer accounted for <50% of the study population, (2) studies that focused on telehealth care only and did not include an electronic or web-based intervention (eg, studies that evaluated remote sessions with a clinician via video link), and (3) studies in which apps that were used to track activity could be linked to a wearable device but did not provide any other support.

### Search Strategy

A search of 6 databases—PubMed, Web of Science, CINAHL, MEDLINE, SPORTDiscus, and PsycINFO—was carried out on April 17, 2023, via EBSCO, using a list of key terms focusing on 3 distinct categories: the intervention characteristics (eg, web-based, internet, app or application, remote, and digital), physical functioning (eg, activity, exercise, training, movement, and athletics), and the population of interest (eg, patients with lung cancer or survivors of lung cancer and patients with cancer). These were amalgamated using Boolean operators to formulate a comprehensive search string. The full list of search terms is presented in Textbox 1. Other sources, such as references of included records, were also searched.

**Groups of keywords used in the search strategy.**

**Search terms**

lung cancer patient* or lung cancer surviv* or lung canceror lung neoplasm* or lung tumor* or lung tumour* or *lung adenocarcinoma* AND *physical activity* or *exercise* or *training* or physical function* or *mobility* or *rehabilitation* or *prehabilitation* or *physical fitness training* or *physical rehabilitation* or *physical recovery* or *mobility training* AND *mobile applications* or *mobile apps* or *mobile phone apps* or *phone apps* or *smartphone apps* or *smartphone applications* or *web apps* or *web applications* or *mhealth* or *m-health* or *ehealth* or *e-health* or *online support system* or *web-based technology* or *app* or *apps* or *software app* or *cell phone apps* or *cellular phone apps* or *mobile technologies* or *mobile devices* or *smartphones* or *technology-enabled care services* or *interactive apps* or *telemedicine* or *virtual medicine* or *interactive consultative services* or *Web based tool* or *activity tracker* or *fitness tracker* or *physical fitness tracker* or *technology enabled care services*

### Data Collection and Analysis

#### Selection of Studies

Of the 794 papers identified through the database search, 213 (26.8%) duplicates were removed, leaving 581 (73.2%) papers. Two reviewers (SK, PRW, CH, or ZD) screened the titles and abstracts of these 581 papers, excluding 544 (93.6%) in the process and retaining 37 (6.4%). Of these 37 papers, a further 7 (23%) were excluded because they were conference abstracts. A full-text screening of the remaining 30 papers was then conducted, and 22 (73%) were excluded, leaving 8 (27%) for final inclusion. Any discrepancies identified by the reviewers during the screening process were resolved by discussion with a third member of the review team. The screening process is outlined in the PRISMA diagram (Multimedia Appendix 1).

#### Data Extraction

Data were independently extracted from the included papers by the lead author (SK) using a data extraction template developed for the purpose of this review. The extracted data were reviewed by the coauthors (CH, ZD, and PRW), and discrepancies were resolved. In case of missing study data, we attempted to contact the corresponding authors to obtain the required information. Three authors were contacted [30-32], but none replied. However, these papers were not excluded because they still met the inclusion criteria and reported on some of our objectives. We extracted the data shown in Textbox 2.

**Extracted data.**

**Study characteristics**

Authors, year of publication, title, country of study, year of study, study objective, overall study design, recruitment method, sample size, participant age range, sex, and study duration

**Intervention characteristics**

Technology product used, setting, intervention details, and exercise details (type, frequency, intensity, and duration)

**Outcome measures of interest**

Impact on physical functioning, impact on HRQoL, and user acceptability

**Other outcomes of interest**

Impact on clinician time, user acceptability or satisfaction, safety features, and cost impact

### Critical Appraisal

#### Risk of Bias

Two reviewers (SK and ZD) independently assessed the risk of bias of each included study and confirmed agreement. The JBI checklists [33] were used to assess the methodological and reporting quality of the included studies.

#### Quality Appraisal

The Grading of Recommendations Assessment, Development, and Evaluation approach was adopted to assess the quality of the evidence used to support the synthesized findings [34,35].

### Data Synthesis and Analysis

Due to the methodological heterogeneity of the included studies, we were unable to undertake a meta-analysis. Instead, the Synthesis Without Meta-Analysis reporting items checklist was used to aid transparency in the reporting process [36]. This enabled us to report on the key features of the included studies, group the studies, and explain our findings. A narrative synthesis [37] was also undertaken in accordance with the study objectives stated earlier. This allowed us to provide a comprehensive summary of the impact and effectiveness of the interventions identified in the included studies.

## Results

### Overview

**Figure 1:**
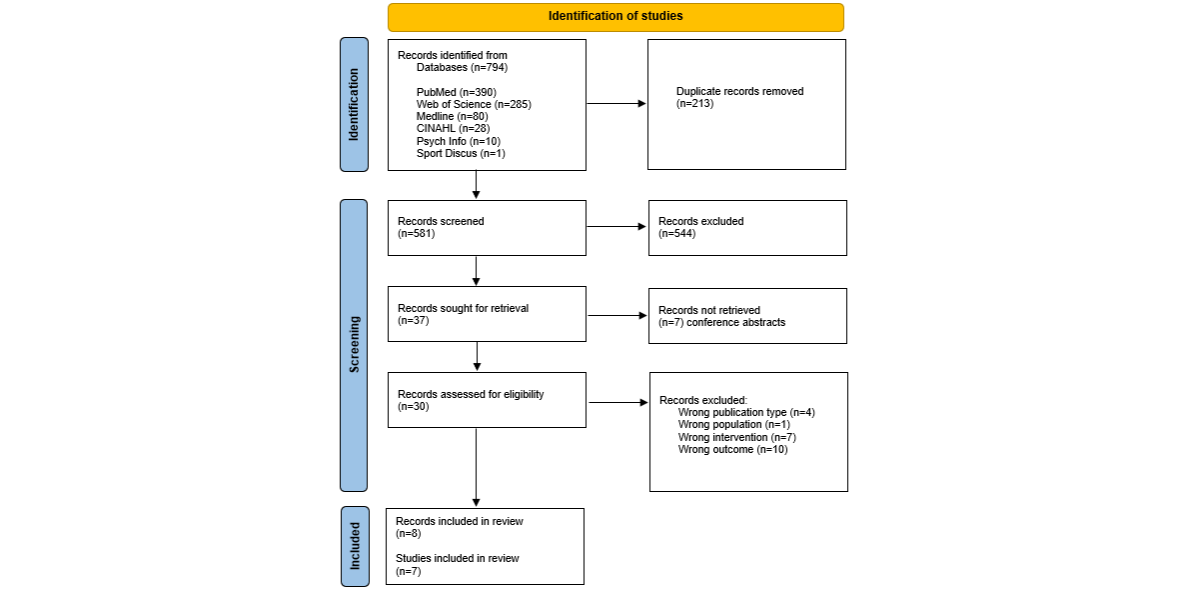
PRISMA (Preferred Reporting Items for Systematic Reviews and Meta-Analyses) flow diagram.

Eight papers were identified as suitable for inclusion. However, 2 (25%) of these 8 papers [30,38] reported on different stages of the same study; therefore, only 7 studies were included in the review. The screening process is outlined in the PRISMA diagram presented in Figure 1.

### Study Characteristics

The included studies were published between 2013 and 2022 across international settings, including the United States [30,38], the Netherlands [39], South Korea [32,40,41], the United Kingdom [31], and Canada [42].

The studies were undertaken between 2010 [30] and 2018 [40]. The aim of the included studies was to develop a technology [31] and test its usability [39], feasibility [31,39,41,42], acceptance [30,31], and efficacy [30,32,38,41]. The studies were primarily quasi-experimental and nonrandomized experimental studies [30,32,39,41,42]. Only 1 randomized controlled trial [40] and 1 cohort study [31] were included. The research methods used were predominantly quantitative (5/7, 71%) [30,32,40-42], with the remaining studies using mixed methods (2/7, 29%) [31,39].

Participants were identified from secondary care settings. The number of study participants ranged from 5 [42] to 100 [41], and their mean ages ranged from 55.1 (SD 8.7) years [41] to 64.6 (SD 6.5) years [30,38]. Overall, there were more female participants (195/340, 57.4%) than male participants (145/340, 42.6%) across 4 (57%) of the 7 studies [30,39-41]; the study by Kadiri et al [31] did not report the sex breakdown of the participants. The intervention duration ranged from 6 to 12 weeks; the study by Kadiri et al [31] did not report the intervention duration, but it was clear that the intervention was delivered pre- and postoperatively with a study duration of ≤18 months. The characteristics of the included studies are detailed in Table 1.

**Table 1:** Characteristics of the included studies.

Authors, year; country	Title	Year of study	Study objectives	Study design	Recruitment method	Sample size (n)	Age (y), mean (SD)	Sex (n)	Lung cancer stage	Intervention duration	Risk of bias	Quality appraisal score
Hoffman et al [38], 2013; United States	Too Sick Not to Exercise Using a 6-Week, Home-Based Exercise Intervention for Cancer-Related Fatigue Self-Management for Postsurgical Non–Small Cell Lung Cancer Patients	2010	To evaluate the feasibility, acceptability, safety, and changes in study end points of a home-based exercise intervention to enhance perceived self-efficacy for cancer-related fatigue self-management for persons after thoracotomy for NSCLC^a^ transitioning from hospital to home	Quasi-experimental non randomized experimental study; first 6 weeks after discharge (quantitative)	Potential participants were identified during clinical appointments while undergoing diagnostics to confirm a potential diagnosis of NSCLC	7	64.6 (6.5)	F^b^=5, M^c^=2	I, II, or III	6 wk	Low	Moderate
Hoffman et al [30], 2014; United States	Virtual Reality Bringing a New Reality to Postthoracotomy Lung Cancer Patients via a Home-Based Exercise Intervention Targeting Fatigue While Undergoing Adjuvant Treatment	2010-2011	To investigate the feasibility, acceptability, and preliminary efficacy of an exercise intervention for postthoracotomy patients with NSCLC to include those initiating and completing adjuvant therapy	Quasi-experimental nonrandomized experimental study; weeks 7 to 16 after discharge (quantitative)	Participants from phase 1 were asked whether they would like to participate in phase 2	—^d^	—	—	—	10 wk	Low	Moderate
Groen et al [39], 2017; Netherlands	Supporting Lung Cancer Patients With an Interactive Patient Portal: Feasibility Study	2014	To evaluate the feasibility and usability of the patient portal and generate preliminary evidence on its impact	Quasi-experimental nonrandomized experimental study (mixed methods)	Patients were approached by letter, followed by a telephone call from the researchers to discuss participation and check further eligibility criteria	37	59.6 (8.4)	F=16, M=21	I, II, or III	4 mo	Low	Moderate
Ji et al [40], 2019; South Korea	Mobile Health Management Platform–Based Pulmonary Rehabilitation for Patients With Non–Small Cell Lung Cancer: Prospective Clinical Trial	2017-2018	To examine the outcome of home-based pulmonary rehabilitation regarding exercise capacity, dyspnea symptoms, and QoL^e^ in adult patients being treated for NSCLC; primary end points were pulmonary function parameters, and the secondary end point was QoL	Randomized controlled trial (quantitative)	Participants identified from the outpatient department of a single tertiary hospital	64	60.50 (9.80) in fixe d-interactive exercise group; 57.97 (10.10) in fixed exercise group	Fixed exercise group: F=45, M=21; fixed-interactive exercise group: F=51, M=24	I-IV	12 wk	Low	Moderate
Kadiri et al [31], 2019; United Kingdom	Fit 4 Surgery, a Bespoke App With Biofeedback Delivers Rehabilitation at Home Before and After Elective Lung Resection	NR^f^	To develop a bespoke pulmonary rehabilitation app and test its feasibility and acceptability to patients undergoing lung resection surgery	Cohort study (mixed methods)	Patients deemed eligible for curative lung cancer surgery, based on British Thoracic Society guidelines, were referred by the multidisciplinary teams to the regional thoracic surgery unit, where potential patients were identified	31	64 (12)	NR	NR	Unclear	Low	Moderate
Park et al [41], 2019; South Korea	Mobile Phone App–Based Pulmonary Rehabilitation for Chemotherapy-Treated Patients With Advanced Lung Cancer: Pilot Study	2016	To determine the feasibility and efficacy of smartphone app–based pulmonary rehabilitation on exercise capacity, symptom management, and QoL in patients with advanced lung cancer undergoing chemotherapy	Quasi-experimental nonrandomized experimental study (quantitative)	Consecutive patients with histologically diagnosed advanced NSCLC were identified	100	55.1 (8.7)	F=54, M=46	II-IV	12 wk	Low	Moderate
Coats et al [42], 2020; Canada	Feasibility of an Eight-Week Telerehabilitation Intervention for Patients With Unresectable Thoracic Neoplasia Receiving Chemotherapy: A Pilot Study	2014	To investigate the feasibility, adherence, and satisfaction of a home-based telerehabilitation program with acquisition of real-time physiological parameters in patients with unresectable thoracic neoplasia receiving chemotherapy and to explore its effects on patients’ functional capacity	Quasi-experimental nonrandomized experimental study (quantitative)	5 consecutive eligible patients diagnosed with unresectable thoracic neoplasia and receiving chemotherapy were recruited	5	62 (7)	F=2, M=3	NR	8 wk	Low	Moderate
Yang et al [32], 2022; South Korea	Evaluation of a Smart After-Care Program for Patients With Lung Cancer: A Prospective, Single-Arm Pilot Study	2015	To evaluate the efficacy of a remote health care program for patients with lung cancer: the Smart After-Care Program	Quasi-experimental nonrandomized experimental study (quantitative)	NR	50	58.3 (11.9)	F=22, M=28	I-IV	12 wk	Low	Moderate

^a^NSCLC: non–small cell lung cancer.

^b^F: female.

^c^M: male.

^d^Not applicable.

^e^QoL: quality of life.

^f^NR: not reported.

### Quality Appraisal

The risk of bias for all studies was low, and quality appraisal scores were moderate (Table 1).

### Intervention Characteristics

The review identified 7 technologies that had been studied in people with lung cancer: 4 (57%) mobile apps [31,32,40,41] and 3 (43%) web applications [30,38,39,42]. Of the 3 web applications, 2 (67%) used a gaming console to deliver part of the exercise prescription [30,38,42].

The interventions were delivered or accessed from various settings: web based [31,32,39,40], home based [30,38,42], or a combination of outpatient department and home based [41].

The frequency, intensity, time, and type formula [43] was used to extract key components of the exercise prescription of each study. Only 5 (62%) of the 8 papers reported the full details of the exercise prescription according to the frequency, intensity, time, and type formula [22,30,31,38,41].

The papers that did not provide a detailed exercise prescription provided more general information and recommendations regarding physical activity [32,39]; alternatively, the interactive app would support the participant to edit the frequency, intensity, and duration of the exercise [40]. Where the technology included an interactive patient portal, it was noted that the physical activity support program that was incorporated in the portal was only used by one-third of the participants [39].

The intervention details are summarized in Table 2.

**Table 2:** Intervention characteristics.

Authors, year	Technology product used	Setting	Intervention details	Exercise details					
				Type	Frequency		Intensity		Time
Hoffman et al [30,38], 2014 and 2013	Nintendo Wii Fit Plus fitness game	Home based	Light-intensity exercise intervention using a game console	Light-intensity walking and balance exercises	Walking: daily for 5 days in week 1, then every day; balance exercises: 5 days per week		Walking: comfortable and self-paced; balance exercises: <3.0 METs^a^		Walking: started at 5 min/d for 5 days during week 1 and was anticipated to increase by 5 min/d each week with the goal of walking 30 min/d during week 6; balance exercises: duration not reported; based on a gaming format and scoring system
Groen et al [39], 2017	MyAVL interactive portal	Online	Web-based patient portal that included physical activity advice	—^b^	—		—	Used a computerized system that provided advice depending on nutritional status; possible contraindications for physical activity; treatment phase; tumor type (breast or lung cancer); whether the patient is participating in a supervised exercise program, and if yes, whether additional information on physical activity is desired	
Ji et al [40], 2019	efil breath app	Online	Personalized pulmonary rehabilitation program using mobile apps: 1 app included fixed exercises, and another app included interactive exercises; a patient monitoring website was also used	Walking and exercises	—	—			The fixed exercise group used the fixed exercise program for 12 weeks; there were 6 levels of walking distance: 600 m, 1200 m, 1800 m, 2400 m, 3000 m, and 3600 m; when the user achieved a fixed walking distance within a day and 14 times in total, the app increased the walking distance to the next level; the interactive exercise group used the fixed exercise program for 6 weeks and then switched to the app with interactive exercises for the next 6 weeks; the interactive exercise used 12 levels; initial walking intensity was set to 80% of the maximum walking speed; once initiated, a metronome in the app was used to help guide the walking speed of the patient; the level of exercise was then adjusted according to the modified Borg scale
Kadiri et al [31], 2019	Fit 4 Surgery app	Web based	Mobile exercise app that included 10 exercises based on the lung cancer “Rehabilitation for Operated Lung Cancer” surgery program	10 exercises: upper and lower limb, aerobic and strength based	Patients’ discretion		Target HR^c^ >60% of maximum HR		3 min/exercise=30 min in total
Park et al [41], 2019	Smart Aftercare app	Outpatient department and home based	Pulmonary rehabilitation program using a smartphone app	Stretching exercise, aerobic exercise, and muscle strengthening	3 times per week		Target HR 70% of HR reserve plus resting HR; oxygen saturation >88%		30-60 min
Coats et al [42], 2020	TELE_RP_ (telerehabilitation program)	Home based	Telerehabilitation program using the eChez-Soi telerehabilitation platform	Exercise ball and elastic bands to exercise upper limbs, as well as wall squats and lunges for lower limbs; cardiovascular exercise with Xbox dance mat and Wii balance board	3 sessions per week, each lasting 75 min, for 8 wks; 15 supervised and 9 unsupervised		SpO_2_^d^ >88%; cardiovascular exercise at moderate intensity; target HR 60%-80% of the VO_2peak_^e^		Repetitions increased according to patients’ tolerance until 2 sets of 15 repetitions; 20 min of cardiovascular exercise
Yang et al [32], 2022	Smart After-Care app	Web based	Mobile app that provided information about rehabilitation exercises and a healthy diet for patients with lung cancer	Muscle strength using elastic bands, stretching, and breathing	NR^f^		NR		NR

^a^MET: metabolic equivalent of task.

^b^Not applicable.

^c^HR: heart rate.

^d^SpO_2_: peripheral oxygen saturation.

^e^VO_2peak_: peak oxygen uptake.

^f^NR: not reported.

### Outcome Measures

#### Primary Outcome Measures

#### Physical Functioning

All included studies demonstrated some improvement in physical functioning, but the methods of assessment varied. The most common assessment was walking time and walking distance. In the studies by Ji et al [40] and Coats et al [42], participants demonstrated an improvement in the 6-minute walk test and the 6-minute walk distance test, while Yang et al [32] noted an improvement in the 2-minute walk distance test. An increase in walking duration and step count was also observed by Hoffman et al [30], while Kadiri et al [31] noted an increase in the distance covered in the shuttle walk test.

No improvement in muscle strength was noted by Coats et al [42], while Yang et al [32] noted an improvement in lower extremity muscle strength but not in upper extremity muscle strength. Only the study by Hoffman et al [30,38] showed an improvement in balance and cancer-related fatigue, while the study by Ji et al [40] showed an improvement in dyspnea grade.

The study by Groen et al [39] identified no significant improvement in physical activity, but an improvement in vigorous activity over time was noted. Finally, the study by Park et al [41] found that although exercise capacity improved in stable patients, this was not the case in patients with progressive disease.

#### Impact on HRQoL

Almost all studies (6/7, 86%) included in our review reported the impact of the digital health intervention on HRQoL [31,32,39-42].

The tool most commonly used to assess HRQoL was the European Organisation for Research and Treatment of Cancer Quality of Life Core Questionnaire-30. Improvements in symptom scales were observed by Kadiri et al [31] and Park et al [41], improvements in functional scales were observed by Yang et al [32] and Park et al [41], while Coats et al [42] did not observe any significant changes.

Ji et al [40] used the EuroQol visual analog scale score to demonstrate a significant improvement between visit 1 and visit 3, but the EQ-5D scores did not differ between the same time points. The Short Form Health Survey-36 was used in the study by Groen et al [39], but no significant changes in the scores were noted.

#### Secondary Outcome Measures

#### Demand on Clinician Time

The technologies varied with regard to the level of clinician involvement required for implementation and ongoing patient management. The authors attempted to assess the impact on clinician time across the included studies (Table 3). Only the study by Kadiri et al [31] explicitly reported the impact of adopting the intervention on health care professional time: 60 minutes.

**Table 3:** Study outcome measures.

Authors, year	Impact on physical functioning	Impact on HRQoL^a^	Demand on clinician time	Acceptability of technology	User satisfaction	Safety features	Costs
Hoffman et al [30,38], 2014 and 2013	Reduced cancer-related fatigue, improved balance and walking duration, and increased step count	Not assessed	Preoperative teaching, postdischarge call, home visit to set up equipment, follow-up call 24 hours later to assess progress and address queries, home visit at 2 weeks, and weekly calls until week 6; ongoing nurse access via telephone during exercise, and nurse would make home visit if there were safety concerns	All participants strongly agreed that exercising at home was convenient, that the nurse interactions during the telephone calls were helpful, and that they would recommend the program to others undergoing similar surgery	Participants agreed strongly that they had a high level of satisfaction with the exercise intervention, giving it a mean score of 5.8 out of 6, with 6 indicating *agreed strongly*, which exceeded the goal of 4 out of 6	Clinical safety: participants required telephone access while exercising should they need assistance; the nurse was available by telephone and could make a home visit if required; and participants were taught how to maintain a light-intensity dose of exercise and also provided with heart rate monitors	Not assessed
Groen et al [39], 2017	Levels of physical activity did not change significantly, but vigorous physical activity tended to increase over time from median 0 (IQR 0-840) MET^b^ min/wk to median 240 (IQR 0-1140) MET min/wk	SF-36^c^: no significant changes	Recruitment procedures and onboarding	93% (25/27) of the patients found the app easy to use, 56% (15/27) reported that it contributed to a sense of control over their health, and 69% (18/26) indicated that it was a valuable addition to their health care experience	Most of the patients (22/27, 81%) were satisfied with the MyAVL portal, and 77% (20/26) intended to continue using it	Authorization procedures (username, password, and SMS text message authentication)	Not assessed
Ji et al [40], 2019	Comparison between pre- and postintervention results; for all participants in both groups, the 6MWD^d^ test performance improved significantly from visit 1 to visit 3 (from mean 433.43, SD 65.60 m to mean 471.25, SD 75.69 m; *P*=.001); subjective dyspnea grade measured using the Modified Medical Research Council Dyspnea Scale improved from visit 1 to visit 3 (from mean 0.94, SD 0.66 to mean 0.61, SD 0.82; *P*=.02); no statistical differences were noted between the fixed and fixed-interactive exercise groups	Comparison between pre- and postintervention results: EQ-VAS^e^ score improved significantly from mean 76.05 (SD 12.37) at visit 1 to mean 82.09, (SD 13.67) at visit 3 (*P*=.002); the mean EQ-5D scores were not significantly different between the time points; no statistical differences were noted between the fixed and fixed-interactive exercise groups	Use a central monitoring website to store records and access summary of compliance and patient health status; measure heart rate and SpO_2_^f^ during exercise, as well as the 6MWT^g^ results	Not assessed	PGA^h^ scores measured at visit 3 showed significant improvement over PGA scores at visit 2 (from mean 13.77, SD 3.68 to mean 15.08, SD 3.99; *P*=.01)	The apps were linked to a wearable pulse oximeter via Bluetooth, and activity-related data were sent to the monitoring website; this is a secure database, which ensures that each participating hospital can only access its own patient data	Not assessed
Kadiri et al [31], 2019	Improved shuttle walk test performance	EORTC QLQ-C30^i^ score improvement noted in fatigue, pain, and dyspnea; the global health score at 5 months for the app significantly increased and returned to baseline level	60 min of health care professional time	Considered acceptable by the researchers because patients in the app group managed more sessions during the pre- and postoperative periods compared to those in the rehabilitation group	Participants found the app easy to use, the ability to see oxygen levels and heart rate was motivational, and they liked the variety of exercises; the novelty factor of using the app for exercise was appealing to some patients, and even patients who had good levels of fitness before using the app found it beneficial	Clinical safety: the app collected baseline measurements of SpO_2_ levels and heart rate for safety; a safety notification screen prevented patients with poor compliance from continuing on the app	£16-£34 (US $20.80-$44.20) per patient
Park et al [41], 2019	Significant difference between before and after the patients in the baseline 6MWD test performance according to baseline ECOG-PS^j^ scores; the mean distance covered was 416.8 (SD 55.4) m in patients with ECOG-PS 0, 369.8 (SD 80.3) m in those with ECOG-PS 1, and 305.7 (SD 89.1) m in patients with ECOG-PS 2 (*P*=.04); after pulmonary rehabilitation, the 6MWD test performance had improved significantly: from 380.1 (SD 74.1) m at baseline and 429.1 (SD 58.6) m (*P*<.001) at 6 weeks to 448.1 (SD 50.0) m (*P*<.001) at 12 weeks; patients with stable disease showed significantly improved 6MWD test performance: from 384.2 (SD 74.6) m at baseline and 426.1 (SD 6.5) m (*P*<.001) at 6 weeks to 447.4 (SD 50.4) m (*P*<.001 at 12 weeks; the dyspnea scale, evaluated using the EORTC QLQ-C30, did not show any significant improvement in the patients overall, but patients with stable disease tended to show improvement	Global health score and HRQoL tended to improve in patients, but these results were not statistically significant	Exercise duration and intensity was prescribed by a physician and adjusted at every clinic visit	NR^k^	90% of the participants were satisfied with the service, and 88% would recommend the program to others	NR	Not assessed
Coats et al [42], 2020	No impact on weight, exercise tolerance, or functional capacity; quadriceps muscle function, peak torque, total work, and fatigue index did not change significantly; the 6MWT and the TST^l^ performance were significantly improved (mean 40, SD 20 m; *P*=.01; and mean –3.0, SD 0.2 s; *P*=.05, respectively)	EORTC QLQ-C30 and EORTC QLQ-LC13^m^: no significant changes in scores	15 supervised sessions (supervised by a clinical exercise physiologist or cancer exercise trainer certified by the American College of Sports Medicine) and 9 unsupervised sessions; 85 hours of kinesiologists’ time and 36 hours spent on installation and uninstallation (technicians and engineers) of the telerehabilitation program	NR	Quebec User Evaluation of Satisfaction with Assistive Technology questionnaire used: participants’ ratings ranged from *quite satisfied* to *very satisfied*	A complete clinical evaluation was made at baseline and after the intervention (within 1 week after the last exercise session); continuous data acquisition and recording from physiological sensors during rehabilitation sessions	Not assessed
Yang et al [32], 2022	No significant difference was observed in upper extremity muscle strength, but significant improvements in lower extremity muscle strength were observed, with repetitions increasing from 18 to 22 for the 30-second chair stand test (*P*=.01); a significant improvement was also noted in walking distance in the 2-minute walk distance test (from 185.7 to 195.0 m; *P*=.03)	EORTC QLQ-C30 functional scales: significant improvements were observed in all subsections (*P*<.05); no significant improvements were seen in the symptom scales; no significant differences were observed in the EORTC QLQ-LC13 scores	Exercise instructions and training from rehabilitation specialist at baseline, 6 weeks, and 12 weeks; in addition, at baseline, training was provided on devices, app, and equipment	Acceptable for, and supportive of, patients with reduced pulmonary function after lung cancer treatment; the Smart After-Care Program was found to be particularly useful for patients living far from the hospital (>80 km)	Assessed at 6 and 12 weeks; in the final satisfaction survey, 88% of the patients rated overall satisfaction as *very good* or *good*	NR	Not assessed

^a^HRQoL: health-related quality of life.

^b^MET: metabolic equivalent of task.

^c^SF-36: Short Form Health Survey-36.

^d^6MWD: 6-minute walk distance.

^e^EQ-VAS: EuroQol visual analog scale.

^f^SpO_2_: peripheral oxygen saturation.

^g^6MWT: 6-minute walk test.

^h^PGA: Patient Global Assessment.

^i^EORTC QLQ-C30: European Organisation for Research and Treatment of Cancer Quality of Life Core Questionnaire-30.

^j^ECOG-PS: Eastern Cooperative Oncology Group-Performance Status.

^k^NR: not reported.

^l^TST: timed stair test.

^m^EORTC QLQ-LC13: EORTC QLQ–Lung Cancer 13.

#### Acceptability and User Satisfaction

All included papers reported either user satisfaction [40-42] or both acceptability and user satisfaction [30-32,39], as summarized in Table 3, and demonstrated high levels of acceptability and user satisfaction.

#### Safety Features

The studies reported primarily on clinical safety features of the technology (eg, access to a mobile phone while exercising to contact the study nurse [30,38], heart rate monitoring [30,31,38,40], and monitoring of peripheral oxygen saturation levels [31], as noted in Table 3). Of the 8 studies, 2 (25%) also reported on the technology’s security features to ensure limited authorized access to the app [40] or database [39].

#### Cost-Effectiveness

None of the studies evaluated the cost-effectiveness of the digital health interventions in terms of clinical outcomes within a health economic context. Only the single-center cohort study [31] reported on the cost of implementing the technology, which was estimated to be between £16 (US $20.8) and £34 (US $44.2) per patient.

## Discussion

### eHealth: Benefits and Concerns

eHealth, which encompasses information and communication technology, facilitates remote care delivery and health information transmission, promoting patient involvement, improving care quality, and enhancing accessibility, particularly in remote areas. Despite benefits such as convenience and reduced travel, concerns exist, such as privacy issues, fears of diminished human interaction, and lack of access to technology and internet infrastructure [44]. eHealth holds promise in supporting patient-centered care and empowers patients by enhancing their involvement in their health care [45].

### Principal Findings

To our knowledge, this is the first review that has looked specifically at digital technologies developed for people with lung cancer and their impact on physical functioning and HRQoL. Despite searching across a wide range of databases, we were only able to identify 7 studies for inclusion in our review.

The findings of this review suggest that digital health interventions with an exercise component are acceptable to people with lung cancer because the interventions help them play a more active role in their health care and can positively impact their physical functioning and HRQoL. Various tools were used in the studies to assess HRQoL. The EQ-5D, a questionnaire conventionally used for economic cost analysis, was used in the study by Coats et al [42] to assess HRQoL, and the authors noted that, despite an observed increase in the EuroQol visual analog scale score, the EQ-5D score did not improve. The evidence to support the use of digital exercise interventions remains sparse, and we have drawn on the findings of only a few studies that aimed primarily to develop and assess the feasibility of the intervention used.

Nevertheless, the quality of the studies was high, thereby strengthening the credibility of the review findings. Further research is required to observe the effects of the interventions over a longer period and to explore the potential cost savings associated with using a remote health management mobile app, including a reduction in clinician time or the number of consultations, improved disease management, and the costs of implementing the technologies in routine care, as well as the safety and security risks of the technologies.

### Results in Context

In recent years, there has been a growth in the number of digital health technologies, including mHealth and eHealth, as well as wearable devices that are becoming an integral part of modern health care [46]. The promotion of patient self-management practices facilitated by digital technology has gained attention because it can improve patient engagement and health care delivery [47]. This has become particularly relevant and has intensified in the wake of the pressures placed on the health service during the COVID-19 pandemic [22]. It is anticipated that strategies to encourage patients to self-manage their health behaviors will continue to be integrated into care pathways in the future [48].

In the context of lung cancer, approximately two-thirds of the people with lung cancer have at least 1 other preexisting health condition, and up to half have ≥2 preexisting health conditions [6], making this group more complex to treat because they require a more tailored approach to rehabilitation [49]. We note that the technologies identified in our review either produced an exercise prescription relevant to the individual participant’s capability or gave the patient guidance on exercises they might be able to undertake. As the participants’ exercise tolerance improved, the prescription was updated, affording a bespoke approach to rehabilitation that might be more relevant to people with lung cancer.

This review highlights the benefits of a digital health intervention that included an exercise component and demonstrated improvements in cancer-related fatigue and balance [30,38], walking duration or distance or step count [30-32,38,42], general activity levels, dyspnea [40], and muscle strength for people living with lung cancer [32]. Nevertheless, there are still areas for development; for example, the study by Yang et al [32] found that while there was an increase in lower extremity muscle strength, no change in upper extremity muscle strength was noted, and, in another study [41], for participants with progressive disease, no improvement was observed in exercise capacity or dyspnea scale score. Furthermore, none of the included studies provided a bespoke exercise prescription and demonstrated a positive impact on exercise endurance, muscle endurance, or dyspnea symptoms. This reinforces the need to produce an exercise prescription that is relevant to the participants’ disease status and individual exercise goals. More consideration is required to develop technologies with intelligent algorithms or artificial intelligence that can support persons with lung cancer to input data on their current condition and capability before a safe, bespoke exercise prescription is recommended. This needs to be factored into app development and further research.

Improving HRQoL is a priority goal in supporting people with lung cancer [49]. All studies that assessed the impact of HRQoL reported a positive impact. The direction of the association between HRQoL and improved physical functioning was consistent across most of the studies (n=5, 71%) that reported on both outcomes [31,32,40-42]. Improvements were primarily noted in the global health score as well as the symptom and functional scales, but it is unclear whether these improvements are a direct result of the exercise intervention or improved disease management and control.

This review highlighted the high levels of acceptability and participant satisfaction with the digital intervention. Having a home-based exercise intervention or web-based intervention, such as the technologies highlighted in this review, is advantageous because participants can continue to exercise during chemotherapy treatment when they are usually advised to limit their contact with other people to reduce the risk of infection. Furthermore, the studies that used a game console as part of their exercise program incentivized their participants to use the technology, work out more often, and increase the intensity of the exercise [30,38,42]. Other useful features of the technologies that have been highlighted include the use of a virtual environment for exercising [30,38], dance-based workouts [42], dietary advice [32], and the value of incentivizing participants [30,38,42]. Hence, it is important for future app developers to consider what incentives can be built into the intervention to improve adherence, reduce clinician workload, and empower patients to be more independent regarding their disease management.

It is anticipated that health technologies, such as the ones identified in this review, will enable improved disease management, reduce hospital admissions, and save the UK National Health Service money [31]. However, limited cost analyses have been conducted to date to demonstrate the cost impact on health care services, the health care workforce, and people living with lung cancer. Our review identified only 1 cohort study [31] undertaken at a single research center that reported the cost of the intervention to the health care provider and the estimated time impact on health care professionals.

### Limitations

We reported on evidence available within the published papers. We faced challenges in gathering details regarding missing study data. We made attempts to reach out to the corresponding authors to obtain the necessary information, but we did not receive any response. Furthermore, it is unclear whether any development information not included in the reports of the studies was excluded [30-32]; in other words, important details could have been left out of the study reports, and the reasons for their possible omission are not known.

The studies included in our review were primarily quasi-experimental and nonrandomized studies [30,32,39,41,42]. Only 1 randomized controlled trial [40] and 1 cohort study [31] were included. The studies focused on technology development and feasibility assessment, most frequently for patients undergoing surgery [30-32,39,40]. In addition, the small cell subtype of lung cancer has poorer outcomes than non–small cell subtypes, with a primary treatment focus being to improve HRQoL; this population was not well represented in the included studies. Furthermore, various tools were used to assess physical functioning and HRQoL, making it difficult to draw comparisons across the studies due to their heterogeneity.

### Conclusions

There seems to be a consistent relationship between the use of digital technologies by people with lung cancer and a positive impact on physical functioning and HRQoL. The technologies reported in this review demonstrated high levels of acceptability or user satisfaction and have the potential to support people with lung cancer to manage their health more independently. However, most of the studies (n=5,71%) identified here report on the development and feasibility of the technology. Further multicenter, large-scale research studies using a randomized controlled trial design over an extended period and including a cost-effectiveness outcome measure are required to fully assess the true benefit of adopting eHealth technologies in standard care services for health care providers and people with lung cancer.

## Appendix

Multimedia Appendix 1 PRISMA (Preferred Reporting Items for Systematic Reviews and Meta-Analyses) checklist.

## Abbreviations

HRQoL: health-related quality of life
mHealth: mobile health
PRISMA: Preferred Reporting Items for Systematic Reviews and Meta-Analyses
